# Understanding feeling “high” and its role in medical cannabis patient outcomes

**DOI:** 10.3389/fphar.2023.1135453

**Published:** 2023-05-24

**Authors:** Sarah S. Stith, Xiaoxue Li, Franco Brockelman, Keenan Keeling, Branden Hall, Jacob M. Vigil

**Affiliations:** ^1^ Department of Economics, University of New Mexico, Albuquerque, NM, United States; ^2^ MoreBetter, Ltd., Hyattsville, MD, United States; ^3^ Department of Psychology, University of New Mexico, Albuquerque, NM, United States

**Keywords:** high, cannabis, marijuana, intoxication, cannabidiol, tetrahydrocannabinol, inebriation

## Abstract

**Introduction:** We measure for the first time the associations between subjective patient experiences of feeling “high” and treatment outcomes during real-time *Cannabis* flower consumption sessions.

**Methods:** Our study uses data from the mobile health app, Releaf App™, through which 1,882 people tracked the effects of *Cannabis* flower on a multitude of health conditions during 16,480 medical cannabis self-administration sessions recorded between 6/5/2016 and 3/11/2021. Session-level reported information included plant phenotypes, modes of administration, potencies, baseline and post-administration symptom intensity levels, total dose used, and real-time side effect experiences.

**Results:** Patients reported feeling high in 49% of cannabis treatment sessions. Using individual patient-level fixed effects regression models and controlling for plant phenotype, consumption mode, tetrahydrocannabinol (THC) and cannabidiol (CBD) potencies, dose, and starting symptom level, our results show that, as compared to sessions in which individuals did not report feeling high, reporting feeling high was associated with a 7.7% decrease in symptom severity from a mean reduction of −3.82 on a 0 to 10 analog scale (coefficient = −0.295, *p* < 0.001) with evidence of a 14.4 percentage point increase (*p* < 0.001) in negative side effect reporting and a 4.4 percentage point (*p* < 0.01) increase in positive side effect reporting. Tetrahydrocannabinol (THC) levels and dose were the strongest statistical predictors of reporting feeling high, while the use of a vaporizer was the strongest inhibitor of feeling high. In symptom-specific models, the association between feeling high and symptom relief remained for people treating pain (*p* < 0.001), anxiety (*p* < 0.001), depression (*p* < 0.01) and fatigue (*p* < 0.01), but was insignificant, though still negative, for people treating insomnia. Although gender and pre-app cannabis experience did not appear to affect the relationship between high and symptom relief, the relationship was larger in magnitude and more statistically significant among patients aged 40 or less.

**Discussion:** The study results suggest clinicians and policymakers should be aware that feeling high is associated with improved symptom relief but increased negative side effects, and factors such as mode of consumption, product potency, and dose can be used to adjust treatment outcomes for the individual patient.

## 1 Introduction

Perhaps there is no more widely referenced, yet under-defined term involving the *Cannabis* plant than that of being or feeling “high” (National Academies of Sciences, Engineering, and Medicine, 2017). Merriam-Webster Dictionary (2022) defines high as “intoxicated by or as if by a drug or alcohol” with “intoxicated” defined both as being under the influence of drugs or alcohol to the point of physical or mental impairment or as “excited, elated, or exhilarated.” In the scientific literature, high is almost always used to convey a pejorative concept associated with intoxication, steaming in part from strong evidence that cannabis intoxication can be associated with significant behavioral risks (Hartman and Huestis, 2013; Chihuri and Li, 2020; Preuss et al., 2021), and in part from the altered state of sensorimotor functioning that defines the very concept of feeling high. Healthcare providers, public health officials, and researchers regularly warn the public of dangers from getting high and the threats posed by people who are high or that get high. State and federal laws and regulations are operationalized based on whether or not the phenotypic expression of the *Cannabis* plant variant (e.g., “hemp”) can get a person high, and recent marketing trends often use the phrasing that cannabidiol (CBD) products can offer medicinal benefits without the risk of producing “a high or any disorientation” (e.g., U.S. Pain Foundation, 2021). At the same time, most medical cannabis patients report enjoying the hyper-sensory experience of feeling high (Clem et al., 2020; Lake et al., 2020), often attributing its visceral euphoria to an enhanced state of peacefulness and relaxation (Stith et al., 2018).

To date, the scientific and medical communities have yet to clearly define the subjective experience of feeling high within the context of pharmaceutical applications of the *Cannabis* plant. Aside from general (millennia-old) descriptions of feeling an enhanced sense of euphoria and experiencing alterations in sensory perceptions, cognition, attention, and abstract thought processing (Russo, 2007; National Institute on Drug Abuse, 2021), there has been little scientific interest in how distinct psychological experiences may be correlated with the plant’s ability to improve or impair the average medical patient’s health outcomes. Phytocannabinoids, such as tetrahydrocannabinol (THC) and CBD, interact with numerous receptors (e.g., CB1) throughout the central nervous system, as well as receptors (e.g., CB2) found in the peripheral immune system, including in white blood cells and the spleen (Kendall and Yudowski, 2017; Bie et al., 2018). However, the precise mechanisms by which cannabis effects changes in psychological functioning and clinical outcomes remain elusive, as does the ability to clearly isolate potentially significant changes in psychological functioning from desired clinical outcomes.

Existing research clearly has shown that at least some of the exhilarating, intoxicating, and impairing characteristics of feeling high are driven by the plant’s THC potency levels (Curran et al., 2020; Cuttler et al., 2021). Since the molecule’s discovery by Raphael Mechoulam’s lab in 1964 (Pertwee, 2006), THC has been identified within the scientific and medical communities as one of the primary determinants of feeling high (National Institute on Drug Abuse, 2021), and hence, THC is the singled-out molecule upon which current U.S. *Cannabis* plant legislation is based, with the 2018 Hemp Farming Act (The Farm Bill) arbitrarily defining legal hemp as *Cannabis* plant variants with less than 0.3% THC potency.

While cannabis with hemp-level THC potency has been shown to induce therapeutic benefits (Blessing et al., 2015; Fakhoury, 2016; Seltzer et al., 2020; Vigil et al., 2020), it is not clear that such treatments are optimal for all patients and conditions, especially health impairments whose very definitions are based on aversive percepts. Conditions such as chronic pain, depression, and anxiety are specific, aversive states of a person’s consciousness (Vigil, 2009; Vigil and Stregnth, 2014; Durisko et al., 2016), and therefore, the ability for psychotropics and entheogens, such as the *Cannabis* plant, to alter the individual’s visceral sensations, perceptions, and attention would seem to be integral to the treatment’s ability to improve these types of health conditions. This thesis is supported by findings showing that THC potency levels are stronger predictors of patient symptom relief than are CBD levels (Stith et al., 2019). While the hope may be that cannabis treatments can offer relief without inducing a feeling of being high, an altered state of consciousness in association with symptom relief is not an unusual side effect among conventional medications such as opioids, benzodiazepines, muscle relaxants, and treatments for hyperactivity and attention deficit disorders.

The current study seeks to measure the associations among experiences of feeling high, patient symptom relief, and side effect outcomes during real-time cannabis administration sessions, accounting for plant phenotype, product potency, consumption mode, and dose and testing for differences across health conditions, sex, and age subgroups. We focus on flower products as the most widely used and homogenous product category with comparable percentage-based potency information across products (Stith et al., 2018). Our data come from the largest database of *Cannabis* flower administration sessions in the U.S., collected by the educational mobile software application, Releaf App™, which provides patients the ability to record the symptom relief and side effects generated by their cannabis usage in real time, across product types, consumption modes, doses, and symptoms. In order to better understand what it means for a patient to feel high, we assess pairwise correlations between feeling high and the other 46 side effects available for selection. We then use individual patient-level fixed effects regression models to assess real-time associations between feeling high and patients’ symptom severity and categories of experienced side effects, both negative and positive. We further evaluate which product characteristics are associated with feeling high and analyze whether feeling high remains an independent predictor of symptom relief and side effect reporting even after controlling for plant phenotype, consumption mode, dose, and THC and CBD potencies. We run subgroup analyses by age, gender, pre-app cannabis experience, and for the five most frequently reported symptoms in the data: pain, anxiety, depression, fatigue, and insomnia.

## 2 Methods

### 2.1 Study design

We analyzed previously collected, de-identified data recorded through the educational mobile software app, Releaf App, which was designed to allow cannabis patients to track their symptom relief and side effect experiences over time across cannabis product characteristics, consumption modes, and symptom types. The de-identified, app-user-level data were provided to the research team by the owner of the Releaf App, MoreBetter, Ltd., under a data confidentiality agreement. (MoreBetter, Ltd. is owned by coauthors, Brockelman, Keeling, and Hall.). The patient-entered data were deemed exempt from IRB review by the Institutional Review Board at the University of New Mexico due to their preexistence and anonymous nature.

To use the Releaf App, patients voluntarily download the free app, available for both Android and iOS devices. Before starting a cannabis session, patients are instructed to record the product characteristics, including product type (flower, concentrate, edible, topical, pill, or tincture), and combustion method for “flower” and “concentrate” (joint, pipe, or vaporizer). Optional reporting includes plant phenotype (*C. sativa*, *C. indica*, or hybrid), which is widely used in marketing and consumer purchasing decisions and recorded by most app users, and THC and CBD potency levels, which are much less widely reported as they are typically available only for dispensary-sourced cannabis due to the cost of potency testing. Once the patient records product characteristics for the cannabis product, the product is saved in the app for future selection. To begin recording the effects of their cannabis treatment, patients are first prompted to select a symptom or set of symptoms for treatment and the cannabis product to be used in treatment before recording their starting symptom intensity level for each symptom on an analog scale from 0 to 10. (For flower and concentrate, combustion method must also be selected.) A session, in which one or more symptoms are tracked, begins when the patient records initial consumption and ends when the patient closes out the session and enters a final rating. While a session is active, patients can update their symptom-specific severity levels at any time, record additional doses (e.g., inhalations in the case of flower), and are able to track a variety of side effects, including seventeen negative side effects, nineteen positive side effects, and eleven side effects which are positive or negative depending on the context, e.g., feeling high or hungry. When setting up an account with the Releaf App, patients are encouraged to record basic demographic information, including age and gender.

The original sample consisted of 232,256 symptom-specific treatment events during 10,6801 sessions recorded by 13,539 app users between 6/5/2016 and 3/11/2021. Restricting the sample to those treatment events with positive starting symptom intensity levels, i.e., involving a health condition in need of treatment, reduced to sample to 228,835 treatment events, 103,825 sessions, and 12,910 app users. In addition, we required that a second symptom level was reported within 4 hours following the session inception to ensure an active cannabis treatment event and that assigned treatment effects are proximate to the timing of cannabis consumption–this restriction left a sample of 196,412 treatment events, 89,258 sessions and 12,908 app users. We further restrict the analysis to sessions using flower, which is the most common type of cannabis product in the data, leaving us with 120,023 treatment events in 57,884 sessions recorded by 9,045 app users. We further restrict the sample to sessions with any side effects reported, leaving us with 94,612 treatment events in 42,751 sessions recorded by 7,396 app users. Because not all variables included in our analyses are required reporting, we lose observations depending on the covariates in the analysis. Including plant phenotype reduces the sample to 84,011 treatment events in 37,991 sessions recorded by 6,862 app users and requiring THC and CBD potencies leaves 18,458 treatment events in 8,780 sessions recorded by 2,100 app users. Our most complete specification uses data on 16,480 treatment events in 7,904 sessions recorded by 1,882 app users. In addition to our main analyses, we conduct subgroup analyses by gender (male versus female), by age (40 or less versus over 40), for the five symptoms most prevalent in our data, and by pre-app cannabis experience (“none” or “a little” versus “a lot” or “expert.”). Out of the total 1,882 users, 1,139 users reported demographic information and 1,210 report pre-app cannabis experience.

### 2.2 Variable construction

We focus on two primary sets of outcomes in this study: symptom relief and side effects. Symptom relief is a treatment event-level outcome and the goal of the patient’s cannabis consumption, while side effects (including high) are reported at the session level and are optional reporting for the patient. Therefore, we use treatment events as our sample in our analyses of symptom relief and sessions as our sample in our analyses of side effects. To measure symptom relief, we calculate symptom change as the lowest symptom level reported within 4 hours from session inception minus the starting symptom level. As shown in Table 1 Panel A, the average symptom change was −3.82, with an average starting symptom level of 5.85 and an average minimum symptom level of 2.03. For side effects, we create three dummy variables for whether a patient reported feeling high (a side effect categorized as context-specific), any negative side effect (out of seventeen) and any positive side effect (out of nineteen) at any time during the session. Patients recorded feeling high in 49% of sessions. With respect to other side effects, patients reported one or more negative side effect in 64% of sessions, one or more positive side effect in 95% of sessions, and one or more context-specific side effects in 81% of sessions. Table 2 lists all the side effects, the category of side effect (negative, positive, or context-specific), and their prevalence in the data.

**TABLE 1:** Descriptive statistics.

	Observations	Patients	Mean	N or SD	Min	Max
Panel A: Outcome Variables						
Symptom Change	16,480	1,882	−3.82	(2.21)	−10	0
Starting Symptom Level	16,480	1,882	5.85	(2.09)	1	10
Minimum Symptom Level	16,480	1,882	2.03	(1.90)	0	10
Reporting Feeling “High”	7,904	1,882	49%	4,041	0	1
Any Negative Side Effect	7,904	1,882	64%	5,074	0	1
Any Positive Side Effect	7,904	1,882	95%	7,531	0	1
Any Context-Specific Side Effect	7,904	1,882	81%	6,439	0	1
Panel B: Treatment Variables						
Reporting Feeling “High”	16,480	1,882	51%	8,471	0	1
Dose	16,480	1,882	9.03	(9.33)	1	115
Plant Phenotype						
Hybrid	16,480	1,882	47%	7,820	0	1
*C. indica*	16,480	1,882	30%	5,015	0	1
*C. sativa*	16,480	1,882	22%	3,645	0	1
Combustion Method						
Joint	16,480	1,882	14%	2,355	0	1
Pipe	16,480	1,882	43%	7,091	0	1
Vape	16,480	1,882	43%	7,034	0	1
THC						
% THC	16,480	1,882	18.05	(6.75)	0	30
THC <10%	16,480	1,882	12%	2,031	0	1
THC 10%–20%	16,480	1,882	42%	6,927	0	1
THC 21%–30%	16,480	1,882	46%	7,522	0	1
CBD						
% CBD	16,480	1,882	5.32	(5.32)	0	30
CBD <1%	16,480	1,882	40%	6,668	0	1
CBD 1%–9%	16,480	1,882	34%	5,557	0	1
CBD 10%–30%	16,480	1,882	26%	4,255	0	1
Panel C: Subgroup Variables						
Common symptoms						
Pain	16,480	1,882	32%	5,307	0	1
Anxiety	16,480	1,882	27%	4,507	0	1
Depression	16,480	1,882	9%	1,436	0	1
Fatigue	16,480	1,882	5%	858	0	1
Insomnia	16,480	1,882	5%	856	0	1
Patient characteristics						
Male	12,478	1,139	49%	6,407	0	1
Age<=40	12,478	1,139	57%	7,165	0	1
Experienced	13,075	1,210	57%	6,414	0	1

Notes: Observation counts are either treatment-level (16,480 observations in the main sample) or session-level (7,904 observations in the main sample.) Demographic characteristics are reported at the treatment level. Dichotomous variables are measured {0,1} and are reported in the tables as percentages ranging from 0 to 100, along with the number of sessions reporting “1.” For non-dichotomous variables, standard deviations are reported in parentheses. In addition to “High,” nineteen positive, seventeen negative, and ten context-specific side effects were available for selection.

**TABLE 2:** Side effect categorization, prevalence, and pairwise correlation with feeling “high.”

Side effect	Category	% Sessions reporting	Pairwise correlation with “high”
Active	Positive	7	0.0615*
Chill	Positive	52	0.3109*
Clear	Positive	21	−0.0477
Comfy	Positive	36	0.0820*
Creative	Positive	9	−0.001
Dreamy	Positive	31	0.1258*
Energetic	Positive	11	0.0241
Focused	Positive	21	−0.0255
Frisky	Positive	9	0.0906*
Grateful	Positive	16	0.1394*
Great	Positive	16	0.1140*
Happy	Positive	25	0.1845*
Light	Positive	23	0.0833*
Optimistic	Positive	19	0.1100*
Peaceful	Positive	51	0.0246
Productive	Positive	14	0.0274
Reflective	Positive	20	−0.0014
Relaxed	Positive	60	0.0500*
Tuned	Positive	22	0.1047*
Anxious	Negative	7	−0.006
Clumsy	Negative	4	0.0970*
Confused	Negative	4	0.0753*
Coughing	Negative	16	0.1304*
Dizzy	Negative	8	0.0695*
Dry Mouth	Negative	30	0.2452*
Foggy	Negative	18	0.1325*
Forgetful	Negative	11	0.0714*
Headache	Negative	5	0.0274
Irritable	Negative	5	0.0234
Nausea	Negative	2	0.0249
Paranoid	Negative	3	0.0727*
Rapid Pulse	Negative	3	0.0603*
Red Eyes	Negative	14	0.2014*
Restless	Negative	14	0.1246*
Scattered	Negative	16	0.1941*
Unmotivated	Negative	12	0.0923*
Couchlocked	Context-Specific	15	0.1377*
Distracted	Context-Specific	13	0.1166*
High	Context-Specific	49	1.0000*
Hungry	Context-Specific	20	0.0964*
Silly	Context-Specific	8	0.1557*
Sleepy	Context-Specific	24	0.0914*
Talkative	Context-Specific	10	0.0578*
Thinky	Context-Specific	15	0.1069*
Thirsty	Context-Specific	29	0.2043*
Tingly	Context-Specific	25	0.2257*
Visuals	Context-Specific	9	0.1848*

Notes: Table includes data from 7,904 sessions. Side effects were categorized as positive, negative, or context-specific by the authors. We report Bonferroni-adjusted Pearson’s correlation coefficients ρ with *’s indicating a statistical significance level of at least 0.01.

Panel B of Table 1 describes the treatment variables. In our analyses of symptom relief, our primary treatment outcome, we use the sample of symptom-specific treatment events and the associated variables are reported in Panel B, noting that we also include these variables in the side effect analyses and rates of prevalence may vary slightly between samples. For example, feeling high was reported in 49% of sessions in Panel A and is reported in 51% of treatment events in Panel B. In order to control for the quantity of cannabis flower consumed, we include the reported dose in our analyses with nine inhalations or “hits” consumed during the average treatment event. The rest of Panel B breaks out the categories of product and consumption characteristics for which high may proxy. Hybrid product (47%) was the most common plant phenotype and using a pipe (43%) was the most common combustion method. The average THC potency level was 18.05% while the average CBD level was 5.32%.

The variables used to define our subsamples are included in Panel C. In terms of symptom subgroups, 32% of treatment events were for Pain (including the specified symptoms: abdominal, arm or leg, back, cramping, gastrointestinal, headache, joint, menstrual, migraine, muscle, neck, nerve and other); 27% for Anxiety (including anxiety, stress, or agitation/irritation); 9% for Depression; 5% for Fatigue; and 5% for Insomnia. For demographics, among users who reported demographics, 49% of treatment events were recorded by males and 57% were recorded by patients 40 years old or younger. The majority of patients in our sample (57%) report being experienced with cannabis prior to starting use of the Releaf App.

### 2.3 Statistical analysis

This study seeks to examine the association between feeling high and symptom relief and side effects experienced, including the mediating roles of dose, plant phenotype, consumption mode, and THC and CBD potencies. Given the lack of a pre-existing clear definition of what it means to feel high, we first present Pearson correlation coefficients (with a Bonferroni adjustment) between reporting feeling high and the other side effects available in the Releaf App. We then proceed in evaluating whether feeling high is associated with changes in symptom relief and side effect reporting by reporting correlations between feeling high, symptom relief, and side effects, controlling for individual fixed effects for all outcomes and controlling for starting symptom level in our symptom relief regressions as it is naturally correlated with the potential extent of symptom relief. Including individual fixed effects allows us to approximate the within-user difference in symptom relief and side effects between sessions in which the patient reported feeling high and sessions in which they did not report feeling high. We next control for dose (the quantity of cannabis consumed) to ensure that feeling high is not merely capturing a greater quantity of cannabis consumed. As the distribution for dose has a long positive tail, we use a natural log transformation in our analysis to diminish the possibility that outliers are influencing the effect. To further explore what factors may be driving respondents feeling high, we regress high on plant phenotype, consumption method and product potency, controlling for individual fixed effects and the natural log of dose, before evaluating whether the effects of feeling high on symptom relief and side effects disappears after we control for plant phenotype, combustion method, and product potency. We also control throughout for starting symptom intensity levels in our symptom relief regressions, and in all our regressions including product characteristics, the natural log of total dose to capture the quantity of cannabis consumed.

In regressions analyzing the role of plant phenotype, consumption mode, and product potency, we measure *C. indica* and *C. sativa* relative to hybrid strains, joint and vape relative to pipe, THC 10%–20% and THC 21%–30% relative to THC less than 10%, and CBD 1%–9% and CBD 10%–30% relative to CBD of less than 1%. We use categorical variables for THC and CBD potency to capture how products are sold (low, medium, and high levels of THC and CBD) and to allow for non-linearities in the relationships. In order to evaluate the difference in coefficients across gender, age, and pre-app cannabis experience subgroups, we use Wald tests to compare coefficients across regressions. As a robustness check, we also run regressions for our main outcomes controlling for the number of context-specific side effects reported (excluding high) in order to control for side effect reporting behavior, such as the same user recording their side effects in greater detail in some sessions, which might mechanistically increase the likelihood of recording a negative or positive side effect. In all regressions, we cluster the standard errors at the user level to control for heteroskedasticity and arbitrary correlation within users. Analyses were conducted using Stata 15.1.

## 3 Results

Table 2 shows reporting frequencies for the side effects available in the Releaf App along correlations between reporting feeling high and reporting of other individual side effects. “High,” reported in 49% of sessions, is one of the most frequently reported side effects–only “Relaxed” (60% of sessions), “Chill” (52% of sessions), and “Peaceful” (51% of sessions) are reported more often. Among Bonferroni-adjusted correlations statistically significant at the 0.01 level (*p* < 0.01), feeling high has the largest statistically significant positive correlations with feeling “Chill,” “Tingly,” and “Thirsty” and experiencing “Dry Mouth” and “Red Eyes.” “High” is negatively correlated with feeling “Clear,” “Focused,” “Anxious,” “Reflective,” and “Creative,” but these correlations are not statistically significant at the 0.01 level. Connecting to the definitions of high discussed in the introduction, feeling high is statistically significantly correlated with impairment-related side effects such as “Clumsy,” “Confused,” “Dizzy,” “Foggy,” and “Paranoid,” as well as euphoria or exhilaration-related effects like “Happy,” “Grateful,” “Great,” and “Optimistic.” The results in Table 2 flesh out what is meant by feeling high among patients in our sample and show that the sensations reported by patients in our sample relate directly to common definitions of feeling high.

We proceed in Table 3 by evaluating the association between feeling high and the treatment outcomes, symptom change, any negative side effect, and any positive side effect, controlling for starting symptom level in the symptom relief regressions and individual fixed effects in all the regressions. We add in the quantity of cannabis consumed, the natural log of the dose, in the second three columns of Table 3. Reporting feeling high is associated with 0.317 greater within-user symptom relief than experienced in treatment events in which the same user did not report feeling high (*p* < 0.001). This improved symptom relief is offset by a 13.6 percentage point increase in the likelihood of reporting a negative side effect, perhaps partially compensated for by a 6.1 percentage point increase in positive side effect reporting. Positive side effects, while not the treatment goal, may improve the overall patient experience, and therefore, medication compliance. A higher starting symptom level is, as expected, positively correlated with symptom relief. The results controlling for the natural log of dose suggest that the variable feeling high is partially capturing the consumption of larger quantities of cannabis, i.e., a larger dose, as the coefficients on the variable high are smaller with the inclusion of Ln Dose. Ln Dose is independently a strongly statistically significant predictor of greater symptom relief and a higher likelihood of reporting side effects, both negative and positive. The effect of a given percentage change in the natural log of the dose variable can be calculated by multiplying the coefficient on the natural log of dose variable by the natural log of one plus the increase, e.g., $\beta_{dose}*\ln\left(1.1\right)$ for a 10% increase or $\beta_{dose}*\ln\left(2\right)$ for the effect of doubling the dose. Doubling the dose is associated with a 0.212-point increase in symptom relief ( $-0.306*\ln\left(2\right)$ and relatively small percentage point increases of 0.028 ( $-0.041*\ln\left(2\right)$ and 0.024 ( $-0.034*\ln\left(2\right)$ for negative and positive side effects, respectively.

**TABLE 3:** Associations between feeling high and treatment outcomes.

	(1)	(2)	(3)	(4)	(5)	(6)
	Symptom change	Any negative	Any positive	Symptom change	Any negative	Any positive
High	−0.317***	0.136***	0.061***	−0.285***	0.132***	0.057***
	(0.023)	(0.009)	(0.007)	(0.023)	(0.009)	(0.007)
Starting Symptom Level	−0.674***			−0.671***		
	(0.007)			(0.007)		
Ln Dose				−0.306***	0.041***	0.034***
				(0.022)	(0.005)	(0.004)
Constant	0.195***	0.356***	0.819***	0.721***	0.284***	0.759***
	(0.047)	(0.004)	(0.003)	(0.062)	(0.011)	(0.008)
Observations	94,703	42,842	42,842	94,612	42,751	42,751
R-squared	0.378	0.018	0.005	0.385	0.020	0.008
N Users	7,419	7,419	7,419	7,396	7,396	7,396

Notes: All regressions are estimated using an individual-level fixed effects model. Standard errors, clustered at the individual patient level, are shown in parentheses. ***p < 0.001, **p < 0.01, *p < 0.05.

Because feeling high may be correlated with specific product and consumption methods, particularly THC, the results in Table 3 may be only capturing a proxy relationship, in which it is not feeling high that is leading to increased symptom relief and negative and positive side effects, but rather a particular plant phenotype, consumption method, or product potency that is driving the effect. Table 4 reports the effects of plant phenotype, consumption method and product potency on the likelihood of reporting feeling high. Starting symptom level and the natural log of total dose are included in all specifications. Results are session-level as side effects are recorded at the session level rather than being specific to an individual symptom being tracked by the patient. In column 1, the likelihood of feeling high is regressed on the natural log of dose and in columns 2–4, we separately include plant phenotype, combustion method, and THC and CBD levels. All covariates are included in column 4. Plant phenotype is not correlated with feeling high. Relative to the use of pipe or joint, vaping is associated with a significantly lower probability of reporting high as a side effect. As expected, a high THC level is a strong predictor of reporting feeling high, while CBD is not associated with feeling high. The quantity of cannabis consumed (Ln Dose) is, not surprisingly, a strong predictor of feeling high.

**TABLE 4:** Associations between plant phenotype, combustion method, and potency and feeling high.

	(1)	(2)	(3)	(4)	(5)
	High	High	High	High	High
*C. indica*		0.006			−0.002
		(0.007)			(0.017)
*C. sativa*		−0.003			−0.022
		(0.008)			(0.018)
Joint			0.004		0.005
			(0.015)		(0.036)
Vape			−0.089***		−0.122***
			(0.014)		(0.026)
THC 10%–20%				0.124**	0.132**
				(0.045)	(0.046)
THC 21%–30%				0.158***	0.164***
				(0.046)	(0.047)
CBD 1%–9%				0.022	0.033
				(0.020)	(0.022)
CBD 10%–30%				−0.030	−0.030
				(0.022)	(0.023)
Ln Dose	0.061***	0.064***	0.068***	0.078***	0.093***
	(0.006)	(0.007)	(0.007)	(0.013)	(0.014)
Constant	0.374***	0.375***	0.393***	0.217***	0.246***
	(0.011)	(0.013)	(0.011)	(0.048)	(0.041)
Observations	42,751	37,991	40,638	8,780	7,904
R-squared	0.007	0.007	0.010	0.025	0.033
N Users	7,396	6,862	7,046	2,100	1,882

Notes: All regressions are estimated using an individual fixed effects model. *C. indica* and *C. sativa* are relative to Hybrid, THC, categories are relative to THC, between 0% and 10%, CBD, categories are relative to <1% CBD, and Joint and Vape are relative to Pipe. Standard errors, clustered at the individual patient level, are shown in parentheses. ***p < 0.001, **p < 0.01, *p < 0.05.

Table 5 presents the association between feeling high and symptom relief, controlling for independent effects from plant phenotype in Column 1, consumption method in Column 2, THC and CBD in Column 3 and all factors jointly in Column 4. In Column 5, we omit high as a covariate to further explore how feeling high interacts with the covariates in Table 4. Throughout our results, plant phenotype is an insignificant predictor of symptom relief, while using a pipe is significantly predictive of greater symptom relief relative to smoking a joint or vaping. THC and CBD are generally insignificant with THC only becoming marginally significant once the variable for feeling high is excluded. This suggests that feeling high is not simply a proxy for consuming a higher THC product. In fact, none of the included product and consumption method covariates dramatically affects the statistical significance or magnitude of the association between feeling high and symptom relief. In our most conservative model, with the full set of covariates included, sessions in which patients reported feeling high have symptom reductions of −0.295 points on average or a 7.7% improvement in symptom relief relative to the average symptom relief of −3.82.

**TABLE 5:** Effects of feeling high and product and combustion characteristics on symptom relief.

	(1)	(2)	(3)	(4)	(5)
	Symptom change	Symptom change	Symptom change	Symptom change	Symptom change
High	−0.291***	−0.277***	−0.296***	−0.295***	
	(0.024)	(0.023)	(0.037)	(0.040)	
*C. indica*	−0.000			0.007	−0.010
	(0.020)			(0.053)	(0.049)
*C. sativa*	0.028			0.018	0.018
	(0.023)			(0.054)	(0.053)
Joint		0.140**		0.283**	0.284***
		(0.047)		(0.088)	(0.084)
Vape		0.198***		0.298***	0.352***
		(0.055)		(0.069)	(0.066)
THC 10%–20%			−0.000	−0.067	−0.112
			(0.081)	(0.080)	(0.079)
THC 21%–30%			−0.053	−0.079	−0.164*
			(0.084)	(0.086)	(0.083)
CBD 1%–9%			−0.019	−0.018	−0.005
			(0.054)	(0.054)	(0.052)
CBD 10%–30%			0.040	0.037	0.019
			(0.059)	(0.061)	(0.061)
Ln Dose	−0.311***	−0.320***	−0.360***	−0.397***	−0.434***
	(0.024)	(0.024)	(0.037)	(0.041)	(0.040)
Starting Symptom Level	−0.675***	−0.671***	−0.668***	−0.672***	−0.656***
	(0.008)	(0.008)	(0.016)	(0.016)	(0.016)
Constant	0.731***	0.649***	0.928***	0.879***	0.767***
	(0.067)	(0.064)	(0.155)	(0.150)	(0.147)
Observations	84,011	89,701	18,458	16,480	19,411
R-squared	0.391	0.388	0.389	0.397	0.380
N Users	6,862	7,046	2,100	1,882	2,181

Notes: All regressions are estimated using an individual patient-level fixed effects model. *C. indica* and *C. sativa* are relative to Hybrid, THC categories are relative to THC between 0% and 10%, CBD categories are relative to <1% CBD, and Joint and Vape are relative to Pipe. Standard errors, clustered at the individual patient level, are shown in parentheses. ***p < 0.001, **p < 0.01, *p < 0.05.

**FIGURE 1:**
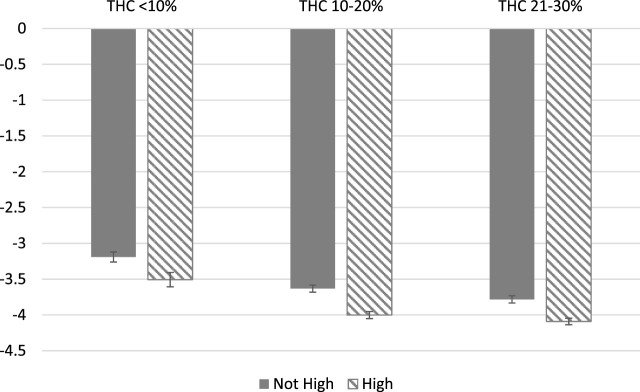
Symptom Relief by High Status and THC levels. Notes: Predicted, covariate-adjusted changes in symptom severity are presented with 95% confidence intervals reported. Covariate-adjusted symptom relief is obtained from an individual fixed effects model controlling for plant phenotype, combustion method, THC and CBD categories, natural log of dose, and starting symptom level.

Figure 1, 2 further demonstrates the relationship between symptom relief, feeling high, and THC level by showing the predicted covariate-adjusted symptom relief by THC levels and whether or not the patient reported feeling high. Consistent with the pattern in Table 4, sessions in which users reported feeling high were associated with greater symptom relief in every THC category.

**FIGURE 2:**
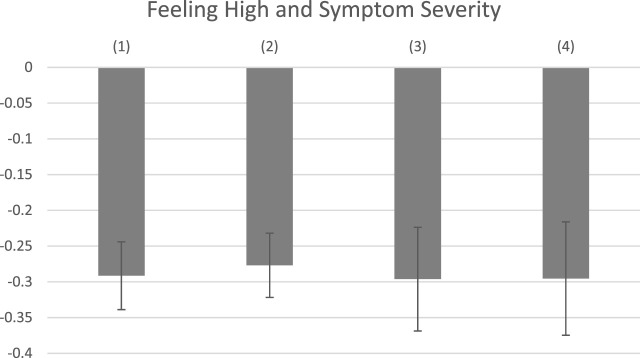
Symptom Relief by Covariate Controls. Note: Covariates-adjusted associations between feeling high and symptom severity are reported with 95% confidence intervals. All regressions control for the log of dosage and starting symptom level. In addition, (1) includes Plant Phenotype; (2) includes Combustion Method; (3) includes THC and CBD levels; (4) includes the full set of product characteristics.

Tables 6, 7 show the effects of reporting feeling high and product and consumption characteristics on negative and positive side effects. Findings suggest that feeling high is associated with higher probabilities of reporting negative and positive side effects. The estimated effects become larger for negative side effects but smaller for positive side effects with the inclusion of product and consumption mode characteristics. Higher THC values are associated with increased negative side effect reporting, but do not appear to be mediating the relationship between feeling high and negative side effects–the coefficients on feeling high increase in magnitude with the inclusion of THC levels and the effects of THC levels on the likelihood of negative side effect reporting are largely unchanged by the inclusion of feeling high, as shown in Columns 4 and 5 of Table 6. We do not find evidence that feeling high is strongly proxying for any of our other covariates with respect to positive side effect reporting, although the negative effect of *C. sativa* and the positive effects of THC increase in magnitude and statistical significance when feeling high is omitted in Column 5 of Table 7.

**TABLE 6:** Effects of feeling high and product and combustion characteristics on negative side effect reporting.

	(1)	(2)	(3)	(4)	(5)
	Any negative	Any negative	Any negative	Any negative	Any negative
High	0.128***	0.131***	0.147***	0.146***	
	(0.009)	(0.009)	(0.016)	(0.017)	
*C. indica*	0.010			−0.017	−0.021
	(0.007)			(0.016)	(0.015)
*C. sativa*	0.001			0.001	−0.009
	(0.007)			(0.017)	(0.016)
Joint		−0.010		0.008	0.023
		(0.013)		(0.039)	(0.037)
Vape		−0.036**		−0.065*	−0.039
		(0.012)		(0.026)	(0.024)
THC 10%–20%			0.048*	0.071**	0.075**
			(0.022)	(0.022)	(0.025)
THC 21%–30%			0.074**	0.100***	0.101**
			(0.028)	(0.028)	(0.031)
CBD 1%–9%			−0.011	0.000	0.000
			(0.017)	(0.017)	(0.018)
CBD 10%–30%			−0.021	0.003	−0.008
			(0.017)	(0.017)	(0.020)
Ln Dose	0.041***	0.048***	0.047***	0.055***	0.032**
	(0.006)	(0.005)	(0.011)	(0.012)	(0.012)
Constant	0.281***	0.285***	0.219***	0.205***	0.403***
	(0.012)	(0.012)	(0.034)	(0.036)	(0.034)
Observations	37,991	40,638	8,780	7,904	9,404
R-squared	0.020	0.021	0.030	0.034	0.005
N Users	6,862	7,046	2,100	1,882	2,181

Notes: All regressions are estimated using an individual patient-level fixed effects model. *C. indica* and *C. sativa* are relative to Hybrid, THC, categories are relative to THC, between 0% and 10%, CBD, categories are relative to <1% CBD, and Joint and Vape are relative to Pipe. Standard errors, clustered at the individual patient level, are shown in parentheses. ***p < 0.001, **p < 0.01, *p < 0.05.

**TABLE 7:** Effects of feeling high and product and combustion characteristics on positive side effect reporting.

	(1)	(2)	(3)	(4)	(5)
	Any positive	Any positive	Any positive	Any positive	Any positive
High	0.058***	0.056***	0.044**	0.044**	
	(0.007)	(0.007)	(0.016)	(0.016)	
*C. indica*	−0.007			−0.029	−0.025
	(0.006)			(0.017)	(0.015)
*C. sativa*	−0.003			−0.041*	−0.044**
	(0.006)			(0.017)	(0.016)
Joint		−0.006		−0.005	0.001
		(0.009)		(0.018)	(0.016)
Vape		−0.012		0.015	0.020
		(0.014)		(0.025)	(0.021)
THC 10%–20%			0.022	0.034	0.042*
			(0.017)	(0.020)	(0.018)
THC 21%–30%			0.001	0.012	0.019
			(0.020)	(0.023)	(0.020)
CBD 1%–9%			0.019	0.015	0.006
			(0.016)	(0.016)	(0.015)
CBD 10%–30%			0.020	0.018	0.013
			(0.018)	(0.019)	(0.017)
Ln Dose	0.034***	0.036***	0.049***	0.046***	0.036***
	(0.005)	(0.005)	(0.010)	(0.010)	(0.008)
Constant	0.763***	0.761***	0.724***	0.731***	0.789***
	(0.009)	(0.010)	(0.023)	(0.025)	(0.020)
Observations	37,991	40,638	8,780	7,904	9,404
R-squared	0.008	0.008	0.011	0.012	0.008
N Users	6,862	7,046	2,100	1,882	2,181

Notes: All regressions are estimated using an individual patient-level fixed effects model. *C. indica* and *C. sativa* are relative to Hybrid, THC, categories are relative to THC, between 0% and 10%, CBD, categories are relative to <1% CBD, and Joint and Vape are relative to Pipe. Standard errors, clustered at the individual user level, are shown in parentheses. ***p < 0.001, **p < 0.01, *p < 0.05.

Tables 8, 9 present our subgroup analyses. To conduct these analyses, we further restrict the analysis sample to patients who reported their age or gender in Table 8 and to patients who reported treating one of the five most common symptoms in our sample in Table 9. As shown in Columns 2 and 3 of Table 8, the association between feeling high on symptom relief is significant for both male and female patients. Male patients show a larger coefficient, but it is not statistically different from the coefficient of the female users (Wald test, *p* = 0.71). Greater variation exists by age group with those over 40 experiencing a smaller and less statistically significant relationship between feeling high and symptom relief (Wald test, *p* < 0.001). The other coefficients in the table suggest fundamental differences between younger and older patients, as combustion methods and THC levels are more relevant for symptom relief among older patients than among younger patients. In our second subgroup analysis in Table 9, we see that feeling high is associated with similar magnitudes of increased symptom relief for Pain, Anxiety, Depression, Fatigue, but not Insomnia, suggesting that feeling high may be too stimulating an experience for sleep induction.

**TABLE 8:** Effects of feeling high and product and combustion characteristics on symptom relief, by gender and age.

	(1)	(2)	(3)	(4)	(5)
	All with demographics	Male	Female	<=40	40+
High	−0.302***	−0.312***	−0.276***	−0.377***	−0.123*
	(0.047)	(0.064)	(0.072)	(0.060)	(0.052)
*C. indica*	0.024	0.031	0.025	0.154	−0.073
	(0.062)	(0.087)	(0.071)	(0.089)	(0.064)
*C. sativa*	0.041	−0.080	0.158	0.056	0.021
	(0.066)	(0.082)	(0.104)	(0.097)	(0.084)
Joint	0.293**	0.293*	0.275*	0.235	0.555***
	(0.097)	(0.133)	(0.135)	(0.130)	(0.105)
Vape	0.307***	0.349***	0.235*	0.242*	0.405***
	(0.079)	(0.103)	(0.111)	(0.119)	(0.099)
THC 10%–20%	−0.099	0.055	−0.272	−0.020	−0.165
	(0.097)	(0.113)	(0.139)	(0.157)	(0.110)
THC 21%–30%	−0.131	−0.122	−0.178	0.064	−0.304*
	(0.101)	(0.120)	(0.168)	(0.153)	(0.120)
CBD 1%–9%	0.013	0.013	−0.026	0.039	−0.047
	(0.063)	(0.085)	(0.081)	(0.089)	(0.076)
CBD 10%–30%	0.021	0.083	−0.080	0.094	−0.051
	(0.072)	(0.093)	(0.107)	(0.104)	(0.104)
Ln Dose	−0.379***	−0.415***	−0.339***	−0.385***	−0.413***
	(0.044)	(0.073)	(0.056)	(0.070)	(0.051)
Starting Symptom Level	−0.680***	−0.691***	−0.672***	−0.685***	−0.683***
	(0.019)	(0.031)	(0.022)	(0.019)	(0.043)
Constant	0.900***	1.012***	0.860**	0.718**	1.104***
	(0.178)	(0.228)	(0.265)	(0.251)	(0.283)
Observations	12,478	6,071	6,407	7,558	4,958
R-squared	0.403	0.432	0.382	0.421	0.390
N Users	1,139	531	608	838	298

Notes: All regressions are estimated using an individual patient-level fixed effects model. *C. indica* and *C. sativa* are relative to Hybrid, THC, categories are relative to THC, between 0% and 10%, CBD, categories are relative to <1% CBD, and Joint and Vape are relative to Pipe. Standard errors, clustered at the individual patient level, are shown in parentheses. ***p < 0.001, **p < 0.01, *p < 0.05.

**TABLE 9:** Effects of feeling high and product and combustion characteristics on symptom relief, by common symptom types.

	(1)	(2)	(3)	(4)	(5)
	Pain	Anxiety	Depression	Fatigue	Insomnia
High	−0.290***	−0.317***	−0.302**	−0.375**	−0.057
	(0.072)	(0.068)	(0.105)	(0.131)	(0.167)
*C. indica*	−0.147*	0.093	0.340**	0.049	0.258
	(0.064)	(0.080)	(0.118)	(0.174)	(0.138)
*C. sativa*	−0.032	0.040	0.035	−0.020	0.259
	(0.081)	(0.074)	(0.118)	(0.128)	(0.245)
Joint	0.156	0.150	0.301	0.477	0.343
	(0.159)	(0.149)	(0.194)	(0.292)	(0.321)
Vape	0.188*	0.161	0.222	0.188	0.192
	(0.088)	(0.121)	(0.201)	(0.273)	(0.237)
THC 10%–20%	−0.049	−0.018	−0.202	−0.047	−0.180
	(0.122)	(0.097)	(0.117)	(0.144)	(0.264)
THC 21%–30%	−0.044	−0.087	−0.232	−0.118	−0.220
	(0.133)	(0.110)	(0.164)	(0.181)	(0.298)
CBD 1%–9%	0.044	−0.041	−0.147	0.043	−0.085
	(0.084)	(0.089)	(0.127)	(0.196)	(0.137)
CBD 10%–30%	0.098	0.038	0.108	−0.041	0.201
	(0.086)	(0.091)	(0.139)	(0.151)	(0.211)
Ln Dose	−0.401***	−0.310***	−0.525***	−0.077	−0.268
	(0.064)	(0.057)	(0.100)	(0.061)	(0.155)
Starting Symptom Level	−0.629***	−0.743***	−0.643***	−0.660***	−0.696***
	(0.025)	(0.023)	(0.033)	(0.070)	(0.064)
Constant	1.157***	0.791***	1.003**	0.439	0.615
	(0.223)	(0.189)	(0.349)	(0.484)	(0.562)
Observations	5,307	4,507	1,436	858	856
R-squared	0.348	0.488	0.412	0.375	0.333
N Users	1,057	1,142	545	357	312

Notes: All regressions are estimated using an individual patient-level fixed effects model. *C. indica* and *C. sativa* are relative to Hybrid, THC, categories are relative to THC, between 0% and 10%, CBD, categories are relative to <1% CBD, and Joint and Vape are relative to Pipe. Standard errors, clustered at the individual user level, are shown in parentheses. ***p < 0.001, **p < 0.01, *p < 0.05.

Table 10 offers suggestive results with respect to the effect of experience on the relationship among symptom relief, product characteristics including THC, and feeling high. Feeling high has similar effects on symptom relief for both experienced and inexperienced users (Wald test, *p* = 0.71). However, the relationship between THC and feeling high does vary between the two groups with the effect of THC larger in magnitude and more statistically significant among inexperienced users than among experienced users and the negative impact of vaping on feeling high only evident among experienced users.

**TABLE 10:** Effects of feeling high and product and combustion characteristics on symptom relief, by pre-app cannabis experience.

	(1)	(2)	(3)	(4)
	Not experienced	Experienced	Not experienced	Experienced
High	−0.289***	−0.311***		
	(0.068)	(0.060)		
*C. indica*	0.005	0.064	0.022	0.025
	(0.066)	(0.105)	(0.025)	(0.029)
*C. sativa*	0.006	0.039	−0.022	0.002
	(0.086)	(0.093)	(0.032)	(0.032)
Joint	0.309*	0.272*	−0.005	−0.028
	(0.151)	(0.127)	(0.051)	(0.060)
Vape	0.336**	0.249*	−0.076	−0.213***
	(0.116)	(0.103)	(0.049)	(0.054)
THC 10%–20%	−0.177	0.038	0.157*	0.081
	(0.113)	(0.160)	(0.067)	(0.053)
THC 21%–30%	−0.140	−0.078	0.197**	0.132*
	(0.136)	(0.157)	(0.072)	(0.058)
CBD 1%–9%	−0.015	0.043	0.018	0.082*
	(0.075)	(0.105)	(0.028)	(0.036)
CBD 10%–30%	−0.003	0.076	−0.064*	0.013
	(0.094)	(0.118)	(0.031)	(0.050)
Log dosage	−0.338***	−0.444***	0.074***	0.111***
	(0.045)	(0.084)	(0.022)	(0.025)
Starting Symptom Level	−0.669***	−0.688***		
	(0.030)	(0.021)		
Constant	0.590*	1.219***	0.279***	0.291***
	(0.255)	(0.244)	(0.067)	(0.070)
Observations	6,661	6,414	6,661	6,414
R-squared	0.377	0.428	0.045	0.032
N Users	526	684	526	684

Notes: The outcome in Columns 1 and 2 is symptom relief; the outcome in Columns 3 and 4 is reporting feeling “high.” All regressions are estimated using an individual patient-level fixed effects model. *C. indica* and *C. sativa* are relative to Hybrid, THC, categories are relative to THC, between 0% and 10%, CBD, categories are relative to <1% CBD, and Joint and Vape are relative to Pipe. Standard errors, clustered at the individual user level, are shown in parentheses. ***p < 0.001, **p < 0.01, *p < 0.05.

For a final robustness check on our results, we rerun our main analyses in Table 11, controlling for the total number of context-specific side effects in order to capture within-user, cross-session behavioral factors influencing the likelihood of reporting side effects. We find little effect on the overall pattern of results, but the number of context-specific side effects reported is correlated with an increased likelihood of reporting feeling high, symptom relief, and positive and negative side effect reporting, leading us to hypothesize that cannabis sessions that are overall more stimulating and encourage greater app engagement—e.g., as proxied by a greater likelihood of reporting side effects—are associated with greater symptom relief and increased side effect reporting.

**TABLE 11:** Associations between feeling high and treatment outcomes, controlling for all covariates and side effect reporting behavior.

	(1)	(2)	(3)	(4)
	High	Symptom change	Any negative	Any positive
High		−0.223***	0.103***	0.034*
		(0.040)	(0.014)	(0.017)
*C. indica*	−0.003	0.023	−0.025	−0.029
	(0.018)	(0.054)	(0.015)	(0.017)
*C. sativa*	−0.019	0.019	0.003	−0.042*
	(0.019)	(0.054)	(0.016)	(0.018)
Joint	0.018	0.273**	0.011	−0.002
	(0.039)	(0.088)	(0.036)	(0.018)
Vape	−0.096**	0.270***	−0.048	0.026
	(0.030)	(0.064)	(0.025)	(0.027)
THC 10%–20%	0.094*	−0.033	0.046*	0.029
	(0.043)	(0.078)	(0.023)	(0.020)
THC 21%–30%	0.135**	−0.044	0.075**	0.006
	(0.046)	(0.080)	(0.027)	(0.023)
CBD 1%–9%	0.018	−0.033	0.017	−0.020
	(0.024)	(0.056)	(0.018)	(0.015)
CBD 10%–30%	−0.035	0.023	0.015	−0.005
	(0.027)	(0.066)	(0.018)	(0.016)
N context specific side effects	0.057***	−0.122***	0.082***	0.019**
	(0.006)	(0.015)	(0.007)	(0.006)
Log dosage	0.076***	−0.370***	0.039***	0.042***
	(0.014)	(0.038)	(0.011)	(0.010)
Starting Symptom Level		−0.671***		
		(0.016)		
Constant	0.212***	1.007***	0.120***	0.731***
	(0.045)	(0.139)	(0.036)	(0.021)
Observations	16,480	16,480	7,904	7,904
R-squared	0.062	0.405	0.093	0.017
N Users	1,882	1,882	1,882	1,882

Notes: All regressions are estimated using an individual patient-level fixed effects model. *C. indica* and *C. sativa* are relative to Hybrid, THC, categories are relative to THC, between 0% and 10%, CBD, categories are relative to <1% CBD, and Joint and Vape are relative to Pipe. Standard errors, clustered at the individual user level, are shown in parentheses. ***p < 0.001, **p < 0.01, *p < 0.05.

## 4 Discussion

The current study extends the previous literature in multiple ways. First, we explore in detail how the experience of feeling high relates to other side effects reported during cannabis consumption and find that it is positively correlated with most side effects. This suggests that feeling high may be associated with increased engagement in the cannabis consumption and app use, as well as both feelings of impairment and feelings of exhilaration and elation. Second, we find that variation in whether or not a person reports feeling high is primarily driven by THC levels and whether a vaporizer was used for combustion. Associated with increased relief from health symptoms and side effects experienced during cannabis consumption. Third, we show that feeling high is associated with increased symptom relief and side effects reporting (both positive and negative) and these relationships remain statistically significant even after controlling for the quantity of cannabis consumed, the characteristics of the flower product (plant phenotype and THC and CBD potencies), and the mode of consumption (pipe, joint, vaporizer). Our results appear generalizable across genders and symptom types, although some heterogeneity exists in these relationships between older and younger patients and across symptom types. The results support the thesis that changes in cognizance that characterize the distinct experience of feeling high may play a statistically and clinically significant role in the medicinal effects of the *Cannabis* plant for some patients.

Among the reported product characteristics, THC potency levels were the only independent predictors of an increased likelihood of reporting feeling high, while vaporizing was associated with a reduced likelihood of feeling high. As in prior work (Stith et al., 2019), THC predicted symptom relief and side effect reporting, but once feeling high was included, THC was no longer predictive of increased symptom relief, although it remained predictive of increased negative side effect reporting. It appears that for most patients in our sample, higher THC levels are only effective at increasing symptom relief if they induce feeling high. However, regardless of whether a patient reports feeling high, higher THC levels appear to be strongly associated with increased side effect reporting. These results suggest that ever-increasing THC levels are not the key to therapeutic benefits. Instead, the seeming drive in the cannabis industry towards ever-increasing THC levels may increase medication non-compliance due to the association between higher THC levels and negative side effect reporting.

These results complicate the common belief that the experience of feeling high is always a negative, tangential side effect of cannabis-based therapies, and instead, support the thesis that feeling high may be a fundamental factor for effective cannabis-based treatment for some patients, perhaps even more relevant than THC potency in determining symptom relief. Therefore, the experience of feeling high may highlight the cost-benefit tradeoffs of therapeutic cannabis use, i.e., the potential costs of increased risk of behavioral/cognitive impairments and the potential benefits of improved symptom management. For some people, the costs of impairment from feeling high may outweigh the perceived benefits, rendering cannabis treatments that make a person feel high a suboptimal choice for such individuals. For chronic health conditions that are not characterized by transient states of aversive percepts, such as metabolic or cellular diseases, feeling high may present indirect detriments (e.g., cognitive and behavioral impairments) or benefits sometimes recorded in the literature as positive side effects, such as increased reported quality of life, behavioral motivations, experienced creativity, ability to accomplish personally fulfilling tasks, and/or improved social relations (Schlienz et al., 2020; Aviram et al., 2021). Among other patients, feeling high may be a direct benefit from consuming cannabis. For health conditions such as chronic pain, depression, and anxiety, experiencing euphoria is the very inverse of the forms of visceral sensations and cognitive percepts that characterize these disorders, meaning the primary goal of the treatments may be to achieve the euphoric state of feeling high and/or the behavioral changes that can results from feeling high (e.g., increased physical activity levels). At a mechanistic level, the therapeutic potential of feeling high may arise from interactions between heuristic feelings of euphoria and the tendency for cannabis to induce attentional distraction (Lundqvist, 2005; Hartman and Huestis, 2013), including the ability to alter the user’s attention away from viscerally unpleasant sensations, thoughts, and memories, and habituation of the startle reflex (Kedzior and Martin-Iverson, 2006; Kedzior et al., 2016).

Given the widespread prevalence of clinical and subclinical medical conditions in the general population, one potential implication from the current results may be that some so-called “recreational” cannabis usage, based on the premise that the user is solely motivated to get high, may be offering medicinal benefits, whether the consumer is aware of such an outcome or not. Survey data shows a strong overlap between medicinal and recreational use among cannabis patients (Pacula et al., 2016), and for many individuals it may be impractical to operationalize the distinction between medical versus recreational cannabis use, as actual usage tends to result in essentially complementary outcomes that cannot easily be independently achieved.

It is interesting that so many users reported feeling high, across THC potency levels, suggesting an important role for additional constituents, e.g., terpenes, in the psychological effects of cannabis consumption (McPartland and Russo, 2012; Fischedick and Elzinga, 2015). Phytochemicals, such as terpenes, have been shown to induce changes (e.g., anesthetic, anxiolytic, sedative) in mood but such studies have been limited by a lack of *in vitro* or *in vivo* data (Behr and Johnen, 2009), mice rather than human subjects (Ito and Ito, 2013); or much higher doses than found in the *Cannabis* plant (Surendran et al., 2021). Our own recent work tested how common combinations of THC, CBD and primary terpenes affected patient outcomes. We found differing effects, even across products with similar THC and CBD levels, suggesting an important role for terpenes and the possibility of a multitude of pharmacodynamics resulting from consuming different cannabis strains with varying phytochemical combinations, or chemovars (Vigil et al., 2023). Precisely how these “entourage” effects arise remains unknown with *in vitro* studies indicating that terpenes do not directly affect cannabinoid receptors, e.g., CB1 and CB2 (Santiago et al., 2019; Finlay et al., 2020). Further supporting a role for cannabinoids beyond THC and CBD, our results throughout showed a strong association between the quantity of cannabis consumed and the effects experienced, regardless of whether the individual felt high. In regressions controlling for both feeling high and the natural log of the dose, doubling the dose of cannabis was associated with three-fourths of the effect of feeling high on symptom relief, with somewhat smaller relative impacts on side effect reporting. Controlling for product characteristics and ingestion methods, including THC, only strengthened the association between the quantity of cannabis consumed and patient outcomes. Adding additional nuance to the relationship, the results showing patients were less likely to report high when vaporizing cannabis or smoking cannabis through a joint, regardless of THC levels, might also support a role for additional constituents in the psychological effects of cannabis consumption beyond THC and CBD (McPartland and Russo, 2012; Fischedick and Elzinga, 2015) as different ingestion methods are associated with different levels of bioavailability for THC (Spindle et al., 2019), CBD, and phytochemicals, such as terpenes (Hädener et al., 2019). Further supporting the role of additional constituents beyond THC, regressions by pre-app cannabis experience suggest that while individuals appear to develop tolerance to THC, other factors, e.g., vaping, become more important determinants of feeling high as experience increases. Placebo effects, arguably more likely among less experienced users, could also explain the closer tie between THC and feeling high among less experienced users. Future research clearly should consider the role of phytochemicals beyond THC and CBD, tolerance, and placebo effects in patient outcomes. Likewise, more research is needed on naturally occurring ratios of major cannabinoids such as THC and CBD, which tend to be expressed asynchronously and can have antithetical pharmacodynamics effects. Because CBD can act as both an inverse agonist (CB_2_ receptors) and as a non-competitive negative allosteric modulator (CB_1_ receptors; Laprairie et al., 2015; McPartland et al., 2015; Pertwee, 2008; Tham et al., 2019; Thomas et al., 2007), it is unclear whether hybridized flower strains and/or synthetic formulates with extracted THC and CBD (e.g., 1-to-1 cannabis products) are aggregating, moderating, or perhaps, *de facto* canceling out each other’s effects.

Despite these important implications, the current dataset has fundamental limitations, particularly due to the lack of randomization of treatment interventions or inclusion of controlled placebo conditions, and the self-selection into app use, both in terms of opting into app use and with respect to recording sessions. For example, our sample is more likely to consist of individuals who anticipate some benefit from cannabis consumption and our sample likely does not include every time an app user consumed cannabis during our sample period. Selection bias could be associated with the possibility of both underestimation and overestimation of the association between reporting feeling high and reported symptom relief. Individuals who tend to feel high and experience significant symptom relief might be satisfied with their cannabis experience and chose not to opt into app use as might those who feel high from cannabis but do not experience symptom relief. Other limitations of the study include the absence of information on the patients’ medical histories, concurrent medication and substance use, and the contexts and settings of cannabis usage. Finally, studies have shown that THC and CBD potency levels reported on product labels can be inaccurate (Vandrey et al., 2015; Bonn-Miller et al., 2017), suggesting the need for improvements in testing and regulatory oversight within the recreational and medical cannabis industries. More comprehensive testing will also enable identification of varying plant chemovars, consisting of unique volumes and ratios of terpenes and even minor cannabinoids, which may facilitate eventual identification of plant variants with reliable psychotropic and clinical effects.

In conclusion, this study finds a novel, positive link between feeling high and symptom relief, even after controlling for THC. However, the benefits in terms of increased symptom relief must be weighed against a statistically and clinically significant increase in negative side effects. Our results suggest a complex relationship between the characteristics of a specific *Cannabis* plant, the consumption process, and therapeutic outcomes. Future studies would benefit from measurement of the mental and physical effects of consuming other, non-cannabinoid phytochemicals that commonly develop in the *Cannabis* plant, such as terpenes, as well as how heat exposure (e.g., through temperature-controlled vaping) and pressure affect their bioavailability and pharmacodynamics. Until we better understand these factors, the medical cannabis available to patients largely will be limited to plant variants developed by for-profit firms that may or may not be formulated for optimal symptom management. Prices remain highly correlated with THC levels, one of the primary factors driving the experience of feeling high, suggesting that the private sector is developing products that make people feel high. Both clinically and policy relevant, the results of this study imply that, for many patients, medical benefits may be optimized by achieving the sensation of feeling high at the minimum necessary THC level. Unfortunately, without further research into the role of other phytochemicals in the plant on symptom management, using commercially available cannabis products to target specific symptoms or develop customized treatments likely will remain elusive.

## Data Availability

The data analyzed in this study is subject to the following licenses/restrictions: Data are available subject to a data confidentiality agreement and licensing fees. Requests to access these datasets should be directed to https://releafapp.com/research/.

## References

[B1] Aviram J. Lewitus G. M. Vysotski Y. Yellin B. Berman P. Shapira A. (2021). Prolonged medical cannabis treatment is associated with quality of life improvement and reduction of analgesic medication consumption in chronic pain patients. Front. Pharmacol. 12, 613805–613814. 10.3389/fphar.2021.613805 34093173 PMC8172141

[B2] Behr A. Johnen L. (2009). Myrcene as a natural base chemical in sustainable chemistry: A critical review. ChemSusChem 2 (12), 1072–1095. 10.1002/cssc.200900186 20013989

[B3] Bie B. Wu J. Foss J. F. Naguib M. (2018). An overview of the cannabinoid type 2 receptor system and its therapeutic potential. Curr. Opin. Anaesthesiol. 31 (4), 407–414. 10.1097/ACO.0000000000000616 29794855 PMC6035094

[B4] Blessing E. M. Steenkamp M. M. Manzanares J. Marmar C. R. (2015). Cannabidiol as a potential treatment for anxiety disorders. Neurotherapeutics 12 (4), 825–836. 10.1007/s13311-015-0387-1 26341731 PMC4604171

[B5] Bonn-Miller M. O. Loflin M. J. E. Thomas B. F. Marcu J. P. Hyke T. Vandrey R. (2017). Labeling accuracy of cannabidiol extracts sold online. J. Am. Med. Assoc. 318, 1708–1709. 10.1001/jama.2017.11909 PMC5818782 29114823

[B6] Chihuri S. Li G. (2020). Direct and indirect effects of marijuana use on the risk of fatal 2-vehicle crash initiation. Inj. Epidemiol. 7 (1), 49–10. 10.1186/s40621-020-00276-9 32921302 PMC7488993

[B7] Clem S. N. Bigand T. L. Wilson M. (2020). Cannabis use motivations among adults prescribed opioids for pain versus opioid addiction. Pain Manag. Nurs. 21 (1), 43–47. 10.1016/j.pmn.2019.06.009 31375419

[B8] Curran T. Devillez H. YorkWilliams S. L. Bidwell L. C. (2020). Acute effects of naturalistic THC vs. CBD use on recognition memory: A preliminary study. J. Cannabis Res. 2 (1), 28. 10.1186/s42238-020-00034-0 33526107 PMC7819319

[B9] Cuttler C. Lafrance E. M. Stueber A. (2021). Acute effects of high - potency cannabis flower and cannabis concentrates on everyday life memory and decision making. Sci. Rep. 1, 13784. 10.1038/s41598-021-93198-5 PMC8253757 34215784

[B10] Durisko Z. Mulsant B. H. McKenzie K. Andrews P. W. (2016). Using evolutionary theory to guide mental health research. Can. J. Psychiatry 61 (3), 159–165. 10.1177/0706743716632517 27254091 PMC4813423

[B11] Fakhoury M. (2016). Could cannabidiol be used as an alternative to antipsychotics? J. Psychiatric Res. 80, 14–21. 10.1016/j.jpsychires.2016.05.013 27267317

[B12] Finlay D. B. Sircombe K. J. Nimick M. Jones C. Glass M. (2020). Terpenoids from cannabis do not mediate an entourage effect by acting at cannabinoid receptors. Front. Pharmacol. 11, 359. 10.3389/fphar.2020.00359 32269529 PMC7109307

[B13] Fischedick J Elzinga S (2015). Cannabinoids and terpenes as chemotaxonomic markers in cannabis. Nat. Prod. Chem. Res. 03. 10.4172/2329-6836.1000181

[B14] Hädener M. Vieten S. Weinmann W. Mahler H. (2019). A preliminary investigation of lung availability of cannabinoids by smoking marijuana or dabbing BHO and decarboxylation rate of THC- and CBD-acids. Forensic Sci. Int. 295, 207–212. 10.1016/j.forsciint.2018.12.021 30638755

[B15] Hartman R. L. Huestis M. A. (2013). Cannabis effects on driving skills. Clin. Chem. 59 (3), 478–492. 10.1373/clinchem.2012.194381 23220273 PMC3836260

[B16] Ito K. Ito M. (2013). The sedative effect of inhaled terpinolene in mice and its structure-activity relationships. J. Nat. Med. 67 (4), 833–837. 10.1007/s11418-012-0732-1 23339024

[B17] Kedzior K. K. Martin-Iverson M. T. (2006). Chronic cannabis use is associated with attention-modulated reduction in prepulse inhibition of the startle reflex in healthy humans. J. Psychopharmacol. 20 (4), 471–484. 10.1177/0269881105057516 16174673

[B18] Kedzior K. K. Wehmann E. Martin-Iverson M. (2016). Habituation of the startle reflex depends on attention in cannabis users. BMC Psychol. 4 (1), 50–11. 10.1186/s40359-016-0158-8 27782849 PMC5080700

[B19] Kendall D. A. Yudowski G. A. (2017). Cannabinoid receptors in the central nervous system: Their signaling and roles in disease. Front. Cell. Neurosci. 10, 294–310. 10.3389/fncel.2016.00294 28101004 PMC5209363

[B20] Lake S. Nosova E. Buxton J. Walsh Z. Socías M. E. Hayashi K. (2020). Characterizing motivations for cannabis use in a cohort of people who use illicit drugs: A latent class analysis. PLoS ONE 15 (5), 02334633–e233518. 10.1371/journal.pone.0233463 PMC7241718 32437443

[B21] Laprairie R. B Bagher A. M Kelly M. E. M Denovan-Wright E. M (2015). Cannabidiol is a negative allosteric modulator of the cannabinoid CB1 receptor. Br. J. Pharmacol. 172, 4790–4805. 10.1111/bph.13250 26218440 PMC4621983

[B22] Lundqvist T. (2005). Cognitive consequences of cannabis use: Comparison with abuse of stimulants and heroin with regard to attention, memory and executive functions. Pharmacol. Biochem. Behav. 81 (2), 319–330. 10.1016/j.pbb.2005.02.017 15925403

[B23] McPartland J. M Duncan M Di Marzo V Pertwee R. G (2015). Are cannabidiol and Δ9-tetrahydrocannabivarin negative modulators of the endocannabinoid system? A systematic review. Br. J. Pharmacol. 172 (3), 737–753. 10.1111/bph.12944 25257544 PMC4301686

[B24] McPartland J. M. Russo E. B. (2012). “Cannabis and Cannabis extracts: Greater than the sum of their parts?,” in Cannabis therapeutics in HIV/AIDS. 10.1300/J175v01n03_08

[B26] National Academies of Sciences, Engineering, and Medicine (2017). “The health effects of cannabis and cannabinoids: The current state of evidence and recommendations for research,” in The health effects of cannabis and cannabinoids. 10.17226/24625 28182367

[B27] National Institute on Drug Abuse (2021). Marijuana drug facts. Retrieved from https://www.drugabuse.gov/publications/drugfacts/marijuana .

[B28] Pacula R. L. Jacobson M. Maksabedian E. J. (2016). In the weeds: A baseline view of cannabis use among legalizing states and their neighbours. Addiction 111 (6), 973–980. 10.1111/add.13282 26687431 PMC5216038

[B29] Pertwee R. G. (2006). Cannabinoid pharmacology: The first 66 years. Br. J. Pharmacol. 147 (Suppl. 1), S163–S171. 10.1038/sj.bjp.0706406 16402100 PMC1760722

[B30] Pertwee R. G. (2008). The diverse CB1 and CB2 receptor pharmacology of three plant cannabinoids: delta9-tetrahydrocannabinol, cannabidiol and delta9-tetrahydrocannabivarin. Br. J. Pharmacol. 153, 199, 215. 10.1038/sj.bjp.0707442 17828291 PMC2219532

[B31] Preuss U. W. Huestis M. A. Schneider M. Hermann D. Lutz B. Hasan A. (2021). Cannabis use and car crashes: A review. Front. Psychiatry 12, 643315–643411. 10.3389/fpsyt.2021.643315 34122176 PMC8195290

[B32] Russo E. B. (2007). History of cannabis and its preparations in saga, science, and sobriquet. Chem. Biodivers. 4, 1614–1648. 10.1002/cbdv.200790144 17712811

[B33] Santiago M. Sachdev S. Arnold J. C. McGregor I. S. Connor M. (2019). Absence of entourage: Terpenoids commonly found in*Cannabis sativa* do not modulate the functional activity of Δ^9^-THC at human CB_1_ and CB_2_ receptors. Cannabis cannabinoid Res. 4 (3), 165–176. 10.1089/can.2019.0016 31559333 PMC6757242

[B34] Schlienz N. J. Scalsky R. Martin E. L. Jackson H. Munson J. Strickland J. C. (2020). A cross-sectional and prospective comparison of medicinal cannabis users and controls on self-reported health. Cannabis Cannabinoid Res. X (X), 548–558. 10.1089/can.2019.0096 PMC8713273 33998852

[B35] Seltzer E. S. Watters A. K. Jr D. M. Granat L. M. (2020). Cannabidiol (CBD) as a promising anti-cancer drug. Cancers(Basel) 12 (11), 3203. 10.3390/cancers12113203 33143283 PMC7693730

[B36] Spindle T. R. Cone E. J. Schlienz N. J. Mitchell J. M. Bigelow G. E. Flegel R. (2019). Acute pharmacokinetic profile of smoked and vaporized cannabis in human blood and oral fluid. J. Anal. Toxicol. 43 (4), 233–258. 10.1093/jat/bky104 30615181 PMC6676961

[B37] Stith S. S. Vigil J. M. Brockelman F. Keeling K. Hall B. (2018). Patient-reported symptom relief following medical cannabis consumption. Front. Pharmacol. 9, 916. 10.3389/fphar.2018.00916 30210337 PMC6121171

[B38] Stith S. S. Vigil J. M. Brockelman F. Keeling K. Hall B. (2019). The association between cannabis product characteristics and symptom relief. Sci. Rep. 9 (1), 2712–2718. 10.1038/s41598-019-39462-1 30804402 PMC6389973

[B39] Surendran S. Qassadi F. Surendran G. Lilley D. Heinrich M. (2021). Myrcene—what are the potential health benefits of this flavouring and aroma agent? Front. Nutr. 8, 699666–699714. 10.3389/fnut.2021.699666 34350208 PMC8326332

[B40] Tham M Yilmaz O. Alaverdashvili M. Kelly M. E. M. Denovan-Wright E. M. Laprairie R. B. (2019). Allosteric and orthosteric pharmacology of cannabidiol and cannabidiol-dimethylheptyl at the type 1 and type 2 cannabinoid receptors. Br. J. Pharmacol. 176, 1455–1469. 10.1111/bph.14440 29981240 PMC6487556

[B41] Thomas A. Baillie G. L. Phillips A. M. Razdan R. K. Ross R. A. Pertwee R. G. (2007). Cannabidiol displays unexpectedly high potency as an antagonist of CB 1 and CB 2 receptor agonists *in vitro* . Br. J. Pharmacol. 150 (5), 613–623. 10.1038/sj.bjp.0707133 17245363 PMC2189767

[B42] U. S. Pain Foundation (2021). Looking to try CBD? Read this first. Retrieved from: https://uspainfoundation.org/blog/looking-to-try-cbd-read-this-first/

[B43] Vandrey R. Raber J. C. Raber M. E. Douglass B. Miller C. Bonn-Miller M. O. (2015). Cannabinoid dose and label accuracy in edible medical cannabis products. JAMA - J. Am. Med. Assoc. 313, 2491–2493. 10.1001/jama.2015.6613 26103034

[B44] Vigil J. M. (2009). A socio-relational framework of sex differences in the expression of emotion. Behav. Brain Sci. 32, 375–390. 10.1017/S0140525X09991075 19825246

[B45] Vigil J. M. Montera M. A. Pentkowski N. S. Diviant J. P. Orozco J. Ortiz A. L. (2020). The therapeutic effectiveness of full spectrum hemp oil using a chronic neuropathic pain model. Life 10, 69. 10.3390/life10050069 32443500 PMC7281216

[B46] Vigil J. M. Stith S. S. Brockelman F. Keeling K. Hall B. (2023). Systematic combinations of major cannabinoid and terpene contents in cannabis flower and patient outcomes: A proof-of-concept assessment of the Vigil index of cannabis chemovars. J. Cannabis Res. 5 (1), 4. 10.1186/s42238-022-00170-9 36755303 PMC9906924

[B47] Vigil J. M. Stregnth C. (2014). No pain, no social gains: A social-signaling perspective of human pain behaviors. World J. Anesthesiol. 4 (3), 18–30. 10.5313/wja.v3.i1.18

